# Effect of Replacing Soybean Meal with Cottonseed Meal or Rapeseed Meal on Growth Performance, Meat Quality, and Metabolome of Raw and Cooked Meat in Finishing Lambs

**DOI:** 10.3390/metabo16060387

**Published:** 2026-06-03

**Authors:** Shuzhen Wang, Xiong Zhao, Wancheng Wu, Guosheng Xin, Xiaodong Chen, Morteza H. Ghaffari, Tao Ma

**Affiliations:** 1 Key Laboratory of Feed Biotechnology of the Ministry of Agriculture and Rural Affairs, Institute of Feed Research of Chinese Academy of Agricultural Sciences, Beijing 100081, China; 18279795398@163.com (S.W.); zhaoxiong2001@163.com (X.Z.); 2 College of Animal Science and Technology, Gansu Agricultural University, Lanzhou 730070, China; 3 Zhongquan Agriculture and Animal Husbandry Co., Ltd., Wuzhong 751500, China; wancheng_wu@163.com; 4 School of Life and Sciences, Ningxia University, Yinchuan 750021, China; gsxin@nxu.edu.cn; 5 College of Animal Science and Technology, Ningxia University, Yinchuan 750021, China; isxiaodong@163.com; 6 Institute for Farm Animal Biology (FBN), 18196 Dummerstorf, Germany

**Keywords:** dietary protein source, Tan sheep, amino acid, metabolome, volatile compound

## Abstract

Objectives: To evaluate the effects of replacing soybean meal (SBM) with rapeseed meal (RSM) or cottonseed meal (CSM) in finishing lamb diets on meat, untargeted metabolomics were used to explore underlying mechanisms of metabolites related to Volatile Organic Compounds (VOCs) between raw and cooked meat. Methods: Twenty-four lambs were fed isocaloric and isonitrogenous diets for 90 days and longissimus thoracic (LT) meat was sampled for quality evaluation. Results: Results showed that growth performance and most traits of meat were unaffected, including feed intake, average daily growth, pH, cooking loss, shear force, most color values, and fatty acids. However, the yellowness (b*) in RSM and CSM meat, as well as Met and Tyr in CSM meat, were increased. Muscle metabolomics identified five different metabolite-associated flavor precursors that varied, including galactose, L-pyrdosine, and 13-octadecenoic acid. In total, 223 VOCs detected in cooked meat showed no major differences among diets. Key flavor compounds, including 1-octen-3-ol and lipid-derived aldehydes, were consistent across treatments. Conclusions: In conclusion, RSM and CSM are viable SBM alternatives, changes in raw meat metabolites do not alter the volatile compounds of cooked meat.

## 1. Introduction

Global demand for animal-derived protein is rising, with meat consumption projected to increase by approximately 102% from 2000 to 2050 [1], driving compound feed demand from 1245 to about 1500 million tons over the same period [2]. Soybean meal (SBM) remains the primary protein source in ruminant diets due to its high protein content and favorable amino acid profile. However, its cost and availability have become increasingly unstable because of market pressures and environmental constraints [3]. As a result, replacing SBM with alternative protein sources has become a priority in both emerging and established livestock systems across various regions, including China [4], Mexico [5], Oman [6], the United States [7], Italy [8], Germany [9], and the United Kingdom [10].

Oilseed meals derived from vegetable oil extraction are especially promising because they do not compete with human food supplies. Rapeseed meal (RSM) has good nutritional content, containing (35–44%) protein content, 12% crude fiber, and a balanced amino acid content that has levels of methionine and cysteine higher than SBM [11,12], while the oligosaccharide content of rapeseed meal is lower than that of soybean meal (5.6–2.0%) [13]. However, the tannins of RSM may bind with proteins or proteases, affecting protein digestion and causing a poor taste. Cottonseed meal (CSM) has a protein content of (34–40%) and 11% crude fiber, and is widely used for ruminants to serve as a substitute for SBM, especially in major cotton-producing regions such as India, China, and the United States [12,14], but the free gossypol retained may cause problems, such as slowing down animal growth [15]. Such problems aside, these two raw materials become promising alternatives to soybean meal [16].

RSM and CSM have been reported to increase the contents of Met and unsaturated fatty acids in meat [17]. These diet-induced alterations in muscle metabolite profiles, particularly in amino acids, intramuscular fat, and fatty acid composition, not only influence key sensory attributes, such as juiciness, tenderness, and mouthfeel [18,19,20,21], but also provide essential precursors for flavor formation. During cooking, Methionine, a bitter-tasting amino acid, can generate bitter compounds when participating in the Maillard reaction with reducing sugars. And unsaturated fatty acids such as oleic acid and arachidonic acid can produce flavor compounds, including (E)-2-octenal, octanal, nonanal, and (E,E)-2,4-decadienal, during thermal oxidation and degradation, thereby influencing the flavor characteristics and consumer acceptance of meat [21,22,23].

Tan sheep (*Ovis aries*), an indigenous breed of Northwestern China, are recognized for their tender meat and mild flavor, with low off-flavor intensity [24]. Previous work has evaluated how feeding strategies or protein ingredient substitutions affect meat quality traits in Tan lambs [24,25]. Yet studies integrating SBM substitution with quantitative meat quality traits and metabolomic profiling across the raw-to-cooked transition remain scarce. This limits mechanistic insight into how different dietary protein sources influence flavor-precursor metabolism, volatile compound generation, and sensory-relevant attributes of lamb meat. Therefore, this study was designed to evaluate the effects of replacing SBM with CSM or RSM on growth performance, longissimus thoracic (LT) meat quality, and muscle metabolomic profiles in lambs, aiming to identify diet-induced metabolic changes related to flavor development and support the use of sustainable alternative protein sources through non-targeted metabolomics of raw and cooked muscle.

## 2. Materials and Methods

### 2.1. Experimental Animals, Design and Diets

Forty-eight weaned Tan lambs (two months old) of similar body weight (21.5 ± 1.0 kg) were used. All lambs were clinically healthy at the start of the trial, randomly assigned to three groups (*n* = 16 per treatment), and fed three diets, which were isocaloric, isonitrogenous, and formulated with SBM, RSM, or CSM as the main protein source, respectively (Table 1). They were fed twice daily at 8:00 am and 6:00 pm, with ad libitum access to clean water. Two sheep were kept and fed in a loop as a duplicate. After grouping lambs into pens, pens were randomly assigned to dietary treatments using a random number generator. For growth performance, the pen was considered the experimental unit. For slaughter and meat quality measurements, one lamb was randomly selected from each pen at the end of the trial to serve as a representative sample. Thus, the pen remained as the experimental unit for statistical analysis to avoid pseudo-replication. Pens were bedded with dry straw and cleaned weekly to maintain consistent sanitary conditions. Environmental temperature and ventilation were maintained within the thermoneutral range (12–20 °C). Health status was monitored daily, and no medical interventions were required during the experimental period.

### 2.2. Growth Performance Measurement

The experiment lasted 75 d, including a 10 d adaptation period. After the adaptation period, the amount of feed offered and refused was recorded daily for each loop to calculate the dry matter intake (DMI). The lambs were weighed at 10 and 75 d of the experiment to calculate the average daily gain (ADG) and feed conversion ratio (FCR) as follows:

DMI (g/d) = Total Feed Intake × DM (%)/Days

ADG (g/d) = (Final Body Weight − Initial Body Weight)/Days

FCR = (Average daily feed intake/ADG)

### 2.3. Meat Quality Measurement

All analyses of meat characteristics were conducted on the longissimus thoracic (LT) samples excised between the last thoracic and the first lumbar vertebrae and posterior to the last lumbar vertebra. All samples were trimmed and subdivided within 1 h postmortem. Some were used for pH and color measurements and the residual material was stored at −20 °C for later analyses. Samples for metabolomics were immediately snap-frozen in liquid nitrogen and stored at −80 °C to preserve metabolic integrity until extraction.

#### 2.3.1. Meat pH and Color Measurement

The pH value was measured and collected in triplicate by directly inserting a calibrated pH meter with automatic temperature compensation (Testo 205, Testo AG, Lenzkirch, Germany) 2 cm deep into the meat. Before measurement, a two-point calibration was used with technical buffer solutions at pH = 4.01 and pH = 7.00, controlled at 25 °C, for measuring pH 45 min after slaughter.

After a blooming time of 45 min, the lightness (L*), redness(a*), and yellowness (b*) of the meat were recorded from three random sites on the surface of the samples. This assessment was performed using a Spectrocolorimeter CR8 (Shenzhen ThreeNH Technology Co., Ltd., Shenzhen, China) in the CIE Lab color space with illuminant D65, a 10° observer angle, and an 8 mm aperture size. The hue angle and chroma were calculated using the following equations: Hue angle = tan^−1^ (b*/a*); Chroma = (a*2 + b*2)^0.5^ [26]. Statistical analysis was conducted using the mean values derived from repeated color measurements. Before each measurements, instrument calibration was performed by using a certified white tile to ensure spectral accuracy.

#### 2.3.2. Cooking Loss and Meat Shear Force Assessment

A total of 20 g LT samples, after removal of the epimysium and any attached fat, were designated as W1, sealed in a bag, and steamed in a water bath at 80 °C until reaching an internal temperature of 70 °C, which was monitored with an electronic thermometer. After steaming, the samples were cooled at room temperature. After drying surface moisture, the weight of the samples was recorded as W2. The percentage of cooking loss was computed based on the formula provided:

Cooking loss(%) = (W1 − W2)/W1 × 100%

Shear force measurements were performed on samples used for cooking loss. Three cylindrical samples measuring 1 × 1 × 0.2 cm were cut parallel to the fiber orientation and tested using an electronic muscle tenderness meter (C-LM3B, Tenova, Harbin, China). The mean of the three replicates was calculated for meat shear force. All muscle specimens were randomly allocated within a single cooking batch, which was utilized for the assessment of both cooking loss and shear force.

#### 2.3.3. Chemical Analysis

Any visible subcutaneous fat, the sarcolemma, and attached fat in LT samples were removed before chemical analysis. The chemical composition of meat, including moisture (method 925.04), fat (ether extract, method 935.38), ash (method 938.08), and protein (crude protein, method 981.10), was determined according to the methods of the AOAC (2005) [27].

#### 2.3.4. The Amino Acid and Fatty Acid Files

The animo acid and fatty acid of 24 LT samples were determined according to a previously described method [28]. A total of 150 mg of ground freeze-dried meat powder after sample preparation was taken to be analyzed using an amino acid analyzer (Model L-8900, Shimadzu, Tokyo, Japan), and then quantified by spectrophotometry at 570 and 440 nm. The fatty acid analysis of samples were performed on a gas chromatograph (Model GC-2030, Shimadzu, Tokyo, Japan) outfitted with a flame ionization detector.

### 2.4. Analysis of Metabolites in Fresh LT

A total of 50 mg fresh LT samples were used, and the sample solution grinded for 6 min (−10 °C, 50 Hz) using a cryogenic tissue grinder to prevent enzymatic degradation, then ultrasonicated and extracted with 1 mL of methanol:water (4:1, *v*/*v*) containing 0.02 mg/mL internal standard solution at 40 kHz for 30 min at 5 °C. After being placed at −20 °C for 30 min, the samples were centrifuged (13,000× *g*, 4 °C) for 15 min, then the supernatant was removed and blown dry under nitrogen for LC–MS/MS analysis to minimize oxidative losses and ensure consistent ionization efficiency. In addition, the quality control samples (QC) were prepared by mixing equal volumes of the supernatants from all samples to monitor the stability of the analyses.

Chromatographic measurements were conducted in both positive and negative electrospray ionization (ESI) modes using a Thermo UHPLC-Q Exactive HF-X system fitted with an ACQUITY HSS T3 column (100 mm × 2.1 mm, 1.8 μm; Waters, Milford, MA, USA). The mobile phase comprised solvent A, which was 0.1% formic acid in acetonitrile:water (95:5, *v*/*v*), and solvent B, consisting of 0.1% formic acid in acetonitrile:isopropanol:water (47.5:47.5:5, *v*/*v*/*v*). A 3 μL sample was injected into a 40 °C column with 0.4 mL/min flow rate. During the analysis period, all samples were stored at 4 °C to prevent temperature-induced shifts in ion response. 

Raw data files were imported into Progenesis QI v3.0 (Nonlinear Dynamics, Waters, MI, USA) software for peak detection and alignment and been analyzed utilizing an online platform provided by Majorbio Biotech Co., Ltd. (Shanghai, China). Metabolic features detected in at least 80% of the samples were retained and normalized. Variables from QC samples with a relative standard deviation (RSD) greater than 30% were eliminated. Metabolites were identified by searching databases, primarily HMDB (http://www.hmdb.ca/ (accessed on 15 July 2024)), Metlin (https://metlin.scripps.edu/ (accessed on 15 July 2024)), and the Majorbio Database.

### 2.5. E-Nose Analysis of Cooked LT

A total of 7–8 g of LT from each lamb were pooled, cooked in an 80 °C water bath for 20–30 min, minced, and homogenized. The homogenized samples were transferred into 15 mL sealed vials and equilibrated at room temperature for 30 min prior to analysis. Electronic nose (E-nose) measurements were performed using a PEN3 system (Airsense Analytics GmbH, Schwerin, Mecklenburg-Vorpommern, Germany) according to Dong et al. (2021) [29]. Instrument parameters were set as follows: cleaning time, 180 s; zeroing time, 10 s; sample preparation time, 5 s; measurement time, 150 s; carrier gas flow rate, 300 mL/min; and injection flow rate, 300 mL/min. Each group had eight replications, where one lamb was one replication. The sensors and corresponding sensitive compounds are listed in Figure of E-nose results.

### 2.6. Volatile Compounds Analysis of LT

The HS-SPME-GC–MS (Agilent, Santa Clara, CA, USA) was used to evaluate volatile compounds in LT samples. Specifically, a 3.0 g sample was placed in a 20 mL headspace vial, which was immediately sealed after adding 2.5 µL of internal standard (Naphthalene-d8 20 µg/mL; n-Pentacosane-D32, 50 µg/mL) and 4 mL NaCl. Volatile constituents were isolated through headspace solid-phase microextraction (HS-SPME) using a SPME Arrow fiber (DVB/Carbon WR/PDMS, 120 μm × 20 mm; Thermo Fisher Scientific, Zwingen, Switzerland). Samples were incubated and extracted at 80 °C, with an incubation time of 20 min and an extraction time of 10 min. The fiber was conditioned at 240 °C for 10 min prior to use, with pre- and post-desorption conditioning times of 2 min each. Desorption was carried out under heat for a duration of 5 min.

For GC–MS analysis, analyses were introduced in split mode (split ratio 10:1) and separated on a VF-WAXms capillary column (25 m × 0.25 mm × 0.2 μm; Agilent CP9204, Santa Clara, CA, USA) before mass spectrometric detection. Helium was used as the carrier gas at a flow rate of 1 mL/min, with a spacer purge flow rate of 3 mL/min and an inlet temperature of 240 °C. The oven program was as follows: initial temperature 40 °C equilibrated for 0 min, increased to 120 °C at 8 °C/min, then to 230 °C at 20 °C/min, and held for 4.5 min for a total run time of 20 min. Mass spectrometry conditions included an electron impact ion source (EI), a 280 °C transfer line, a 230 °C ion source, a 150 °C quadrupole, and an electron energy of 70 eV. Mass spectrometric analysis was performed in full-scan (SCAN) mode over an *m*/*z* range of 35–500, with a scan rate of 3.2 scans/s. The mass resolution was set to 30,000. Identification of volatile compounds was achieved by comparing the mass spectra with the data system library (NIST-2023), gc-orbitrap flavor and fragrances v1.0 and linear retention index. Known false-positive peaks (including noise, column bleed, and derivatization reagent peaks) have been removed from the data matrix, followed by deduplication and peak merging. Data quantification was performed using an internal standard calibration method. ROAV (Relative Odor Activity Value) is an indicator used to measure the contribution of a specific component to the overall odor. If the ROAV of a compound is ≥1, it is generally considered a key odor component, and a higher ROAV indicates a greater contribution to the odor. The ROAV value was determined using the formula given below:

ROAV = (C%A/C%min) × (Tmin/TA) × 100%

where A is compound in the sample to be tested; min refers to the substance with ROAV equal to 100 (generally, the substance with the smallest odor threshold among the defined flavor thresholds is assigned an ROAV of 100); C%min is the peak area of the compound; Tmin is the threshold of the compound, i.e., 100; C%A is the peak area of the compound to be calculated; and TA is the threshold of the compound to be calculated.

### 2.7. Data Calculation and Statistical Analysis

Eight animals were used per treatment group, and each animal was considered as an independent experimental unit for statistical analysis. All growth performance traits and meat quality parameters were analyzed using R (Version 4.3.0) [30]. Prior to statistical testing, the Shapiro–Wilk and Levene’s tests were used to assess normality and homogeneity of variance, respectively. Variables that did not meet normality assumptions were log-transformed to improve distributional characteristics when appropriate. For assumption-compliant data, one-way ANOVA was performed to evaluate differences among the three treatments, followed by Duncan’s multiple range test using the agricolae package, with statistical significance set at *p* < 0.05. When only two groups were compared, pairwise comparisons were conducted using Student’s *t*-test under the same assumption checks. If data still violated normality or homogeneity assumptions after transformation, the Kruskal–Wallis test was used as a non-parametric alternative. 

Metabolomic data were processed using MetaboAnalyst 6.0 (https://dev.metaboanalyst.ca/, accessed on 27 May 2026). Prior to statistical analysis, datasets were inspected for numerical consistency and standard formatting. Metabolites with more than 20% missing values were excluded according to the 80% rule, and variables with near-zero variance were removed. No additional outlier removal was applied beyond these criteria.

For untargeted metabolomics of fresh meat, raw peak intensity data were normalized by median to reduce systematic technical variation, followed by log transformation (log10) to reduce heteroscedasticity and auto-scaling to center variables to zero mean and unit variance. For volatile metabolomics of cooked meat, data were normalized by sum, log-transformed (log10), and subsequently Pareto-scaled, in which each variable was mean-centered and divided by the square root of its standard deviation to retain medium-intensity signals while reducing dominance of high-abundance compounds.

Multivariate analyses included principal component analysis (PCA) for unsupervised pattern recognition and partial least squares discriminant analysis (PLS-DA) for supervised classification, although PLS-DA results were interpreted cautiously and only reported when model validation indicated acceptable predictive performance. Group differences in metabolite profiles were evaluated using permutation-based multivariate analysis of variance (PERMANOVA) with 999 permutations. Statistical significance was assessed based on F-values, R^2^, and permutation-derived *p*-values. Volcano plot analysis was applied to identify differential metabolites between dietary treatments, integrating biological relevance (fold change (FC) > 2) and statistical significance (FDR-adjusted *p* < 0.05).

## 3. Results

### 3.1. Growth Performance of Lambs

Protein sources had no significant effects on final body weight (FBW), DMI, or ADG of lambs (*p* > 0.05; Table 2). The FCR of lambs in the RSM group tended to be lower than those in SBM and CSM groups (*p* = 0.096).

### 3.2. Meat Quality of LT

No significant differences were observed in moisture (*p* = 0.666), protein (*p* = 0.573), fat (*p* = 0.217), ash (*p* = 0.915), cooking loss (*p* = 0.140), shear force (*p* = 0.903), L* (*p* = 0.242), and a* (*p* = 0.325) of the LT samples among groups (Table 3). The b* of the LT samples were higher in CSM (*p* = 0.014) and RSM (*p* = 0.039) than in SBM, whereas no significant difference was detected between the CSM and RSM groups (*p* > 0.05).

Replacing SBM with CSM or RSM had no significant effect on the concentration of each individual fatty acids or total saturated fatty acids (SFA), monounsaturated fatty acids (MUFA), and polyunsaturated fatty acids (PUFA) in the LT samples among groups (*p* > 0.05; Table 4). Similarly, the concentrations of total amino acids (TAA), essential amino acids (EAA), flavor amino acids (FAA), and most of individual amino acids in the LT samples were not different among groups (*p* > 0.05; Table 5). Specifically, the concentration of Met (*p* = 0.020) and Tyr (*p* = 0.037) was higher in the LT sample in CSM group compared with that in SBM group. In addition, the concentration of Ala (*p* = 0.07) tended to be higher in the CSM group compared with that in SBM group.

### 3.3. Metabolites of Fresh LT

**Figure 1:**
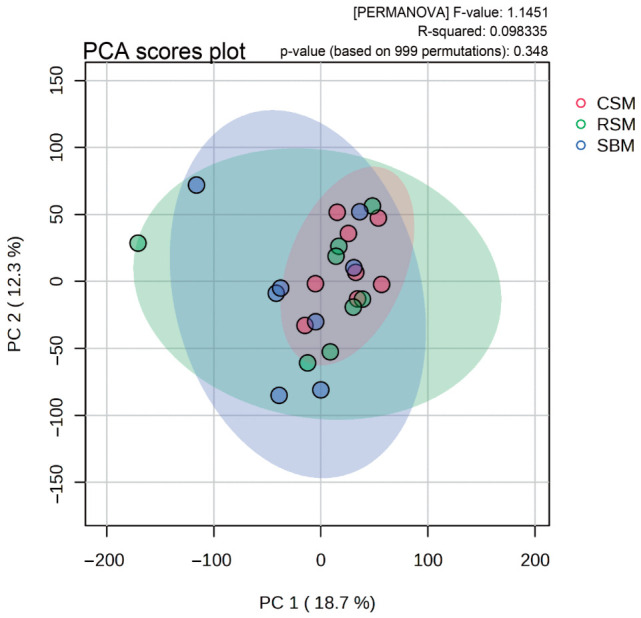
PCA score plot of metabolites in fresh LT across three groups: cottonseed meal group (CSM), rape),eed meal group (RSM) and soybean meal group (SBM); PC1 and PC2 explain 18.7 % and 12.3 % of the total variance, respectively. n = 8 per group, the ellipse indicates the 95 % confidence region. Based on the PCA score plot, PERMANOVA yielded an F-value of 1.1451, an R^2^ value of 0.098, and a non-significant permutation-based *p*-value (*p* = 0.348, 999 permutations).

Across both GC–MS and LC–MS platforms, a total of 1341 metabolites were annotated in fresh LT samples (Supplementary Table S1). Principal component analysis (PCA) performed after ANOVA with Benjamini–Hochberg correction did not show significant group separation. The first two principal components explained 31.0% of the total variance, and PERMANOVA further confirmed that the overall metabolomic profiles were not significantly separated (F = 1.15, R^2^ = 0.098, *p* = 0.348) (Figure 1). However, differential feature metabolites (DFMs) among dietary groups were further screened using volcano plot analysis based on a FC > 2 and a false discovery rate (FDR) < 0.05. In total, five DFMs were identified across pairwise comparisons.

**Figure 2:**
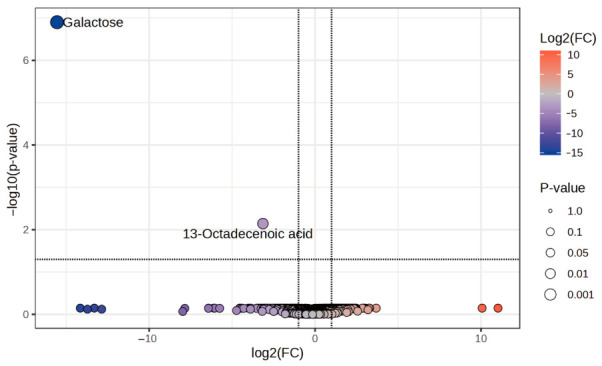
Volcano plot analysis of differential metabolites between the cottonseed meal group (CSM) and soybean meal group (SBM). The horizontal axis represents the log_2_ FC, and the y-axis corresponds to the −log_10_-transformed *p*-values adjusted for the false discovery rate (FDR). The vertical dashed lines indicate the thresholds for |FC| ≥ 2, and the horizontal dashed line represents FDR = 0.05. Metabolites above these thresholds represent significant differences, meeting the criteria of |FC| ≥ 2 and FDR < 0.05. Same as the picture below.

Compared with SBM, CSM exhibited significantly lower abundances of galactose and 13-octadecenoic acid (Figure 2). 

**Figure 3:**
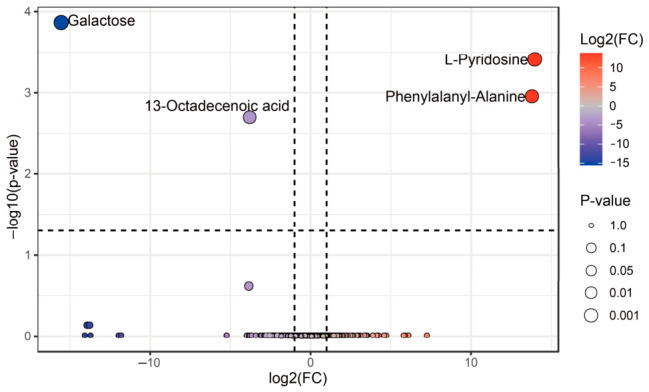
Volcano plot analysis of differential metabolites between the rapeseed meal group (RSM) and soybean meal group (SBM).

Relative to SBM, RSM group showed significantly higher levels of L-pyridosine and phenylalanyl-alanine, while galactose and 13-octadecenoic acid were decreased (Figure 3). 

**Figure 4:**
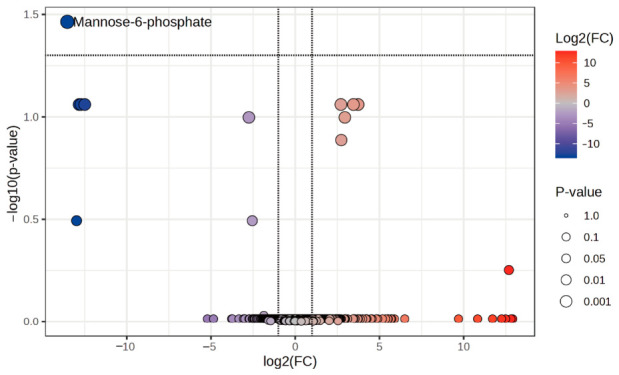
Volcano plot analysis of differential metabolites between the rapeseed meal group (RSM) and cottonseed meal group (CSM).

When comparing the two alternative protein sources, the CSM group displayed a significantly higher abundance of mannose-6-phosphate than the RSM group (Figure 4).

### 3.4. Electronic Nose Analysis and VOC Profiles in Cooked LT Samples

**Figure 5:**
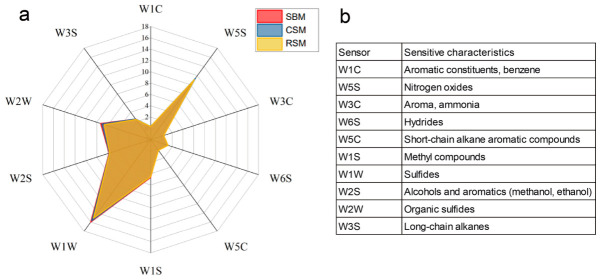
(**a**) E-nose radar plot of SBM, CSM, and RSM for 10 sensors; (**b**) sensitive characteristics relative to E-nose sensors.

Radar plots showed a high degree of overlap in sensor responses among groups, suggesting broadly comparable sensor response patterns (Figure 5a). Notably, slightly higher responses were observed for SBM and CSM compared with RSM in sensors (W1W and W2W), which are reported to be sensitive to sulfur-related and aromatic compounds (Figure 5b).

Volatile Organic Compounds (VOCs) refer to organic chemical substances with a vapor pressure above 0.1 mmHg (13.3 Pa) and a boiling point below 260 °C under normal conditions (20 °C, 101.3 kPa). Based on their chemical structures, VOCs can be further classified into 15 categories: hydrocarbons, acids, esters, alcohols, terpenes, aldehydes, ketones, ethers, amines, phenols, heterocyclic compounds, nitrogen-containing organic compounds, organic sulfur compounds, halogenated hydrocarbons, and other compounds.

**Figure 6:**
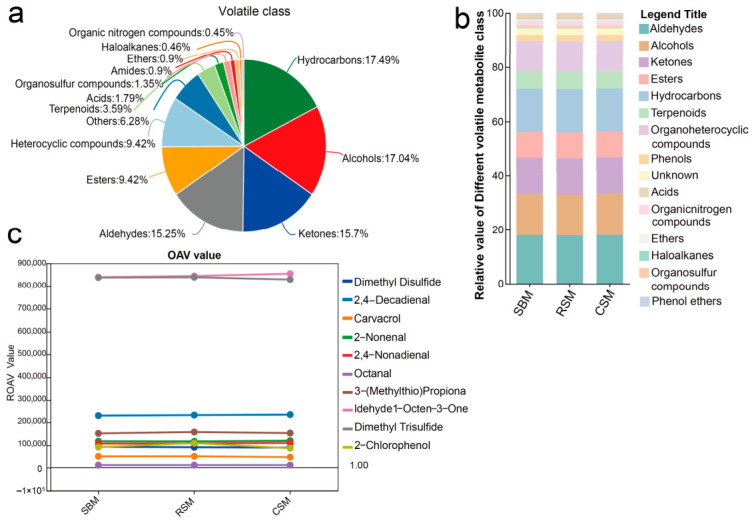
(**a**) Percentage of volatile class in 223 flavor metabolites among the three groups. (**b**) The legend indicates the 15 categories of VOCs identified. (**c**) Line plot depicts the trend of ROAV for key flavor compounds across the three groups (SBM, RSM, CSM).

Through HS-SPME-GS-MS analysis, all VOCs in cooked LT samples were detected and classified. A total of 223 VOCs were tentatively detected in the positive ion mode (Supplementary Table S2). Classification of these compounds revealed that hydrocarbons (17.49%), alcohols (17.04%), ketones (15.70%), and aldehydes (15.25%) accounted for the largest proportions, followed by esters (9.42%) and heterocyclic compounds (9.42%), while terpenes, acids, sulfur-containing, nitrogen-containing, and halogenated compounds were present at lower proportions (Figure 6a). No significant differences were observed at the primary chemical class level (Figure 6b), and one-way ANOVA performed on secondary classifications indicated no significant differences in individually detected VOCs among the three groups (Supplementary Table S3).

Accordingly, subsequent analyses explored differences in ROAVs as a relative ranking metric. ROAV analysis (Supplementary Table S4) highlighted odorants with higher relative ROAV values (ROAV ≥ 1) across all groups, including 1-octen-3-one, dimethyl trisulfide, (2E,4E)-2,4-decadienal, (E)-2-nonenal, (2E,4E)-2,4-nonadienal, 3-(methylthio)propionaldehyde, octanal, 2-chlorophenol, and carvacrol. Line plot visualization showed that 1-octen-3-ol and dimethyl trisulfide consistently exhibited the highest ROAVs across all treatments (Figure 6c).

## 4. Discussion

### 4.1. Growth Performance

Replacing SBM with RSM or CSM as the main dietary protein source did not affect FBW, DMI, or ADG, indicating that animal performance was maintained when diets were formulated to be isocaloric and isonitrogenous. This result aligns with the ability of ruminants to buffer moderate differences in dietary AA patterns through rumen microbial protein synthesis and endogenous N recycling, thereby stabilizing the metabolizable protein supply to peripheral tissues. No significant difference was found in ADG, suggesting the diet with oilseed meals substituting soybean meal can provide equal and balanced nutrition to lamb. The numerical trend toward improved FCR in the RSM group suggests a potential efficiency advantage. Although this effect did not reach statistical significance, small changes in FCR may still have practical relevance in commercial systems. Consistent with this, previous studies in sheep systems have reported that substituting SBM with oilseed meals did not alter intake, ADG, or feed efficiency, including in Santa Inês × Dorper crossbred rams [31], Hu lambs [5], and Dorper × Hu crossbreds [32]. 

### 4.2. Meat Quality

In the current study, replacing SBM with CSM or RSM did not change the proximate composition of LT; moisture, ash, CP, and IMF were similar among treatments and consistent with published values for lamb muscle [33]. The mean SF (43.7 N/cm^2^) indicated a moderate texture and remained below commonly cited consumer acceptability thresholds [34]. Cooking loss and SF were unaffected, indicating that instrumental tenderness was stable across diets. These traits reflect postmortem protein denaturation, myofibrillar shrinkage, and connective tissue effects, the lack of treatment differences suggests that substituting protein sources did not affect the structural determinants of yield and texture under standardized chilling conditions. 

In the present study, dietary effects on instrumental color were limited to an increase in b* in the CSM and RSM groups, while L* and a* remained within comparable consumer-accepted ranges (a* > 9.5, L* > 34) [35]. Meat color primarily reflects myoglobin concentration and heme redox status, which regulate the relative abundance of deoxy-, oxy-, and metmyoglobin [36]. Higher b* values correlate with decreased perceived freshness due to oxidative discoloration, and the elevated b* in CSM and RSM groups suggests weakened pigment stability, which impairs lamb sensory freshness of customers, increases oxidation risk, shortens shelf life, and limits long-distance transportation suitability from marketing perspectives [37,38]. Ingredient-specific minor components of oilseed meals, including lower carotenoid content and differences in antioxidant-active compounds, may influence oxidative balance and metmyoglobin-reducing capacity, while lipid-derived oxidation products can further promote heme oxidation. Although higher L* is positively associated with IMF, which can enhance tenderness and flavor by reducing effective muscle fiber density and supporting flavor development [38], the lack of significant differences in IMF and SF suggests that any diet-related variation in IMF deposition was limited under the conditions of this study.

Replacing SBM with CSM or RSM had negligible effects on the FA profile of the LT, as neither individual FA nor aggregate SFA, MUFA, and PUFA pools differed among treatments. The dominant FA across diets (C16:0, C18:0, C18:1n9t, C18:1n9c, and C18:2n6c) match typical ovine muscle lipid composition [39]. The n-6/n-3 PUFA ratio also remained stable (33–40), indicating that protein source substitution did not alter the balance of essential FA, an attribute relevant to the nutritional value of meat [40]. These outcomes are consistent with previous work, which showed limited effects of alternative plant protein sources on lamb FA composition when diets are formulated to comparable nutrient density [41,42], likely due to rumen biohydrogenation and the absence of targeted lipid supplementation. The stable FA substrate pool also supports the limited differences observed in cooked meat volatile outputs, although oxidation susceptibility may still vary with antioxidant status and pro-oxidant catalysts, warranting measurement of oxidative indices in future work.

The AA profiles of the LT were largely conserved across diets, as TAA, EAA, and FAA did not differ among treatments, indicating that substituting SBM with CSM or RSM had minimal impact on overall muscle AA composition. Nevertheless, the CSM group showed higher Met and Tyr levels, with a tendency toward higher Ala, suggesting that cottonseed-based protein may modestly influence specific precursor pools relevant to flavor development. Alanine is associated with mild sweetness and can contribute to a favorable taste balance in meat [43]. Methionine, despite its potential to contribute bitterness under certain Maillard conditions, is a key sulfur AA precursor that can yield highly odor-active sulfur volatiles (e.g., methional) through Strecker degradation and subsequent reactions, thereby shaping the characteristic cooked-meat aroma [43,44]. Tyrosine participates in Maillard chemistry and can promote the formation of phenolic-like and N-containing heterocyclic compounds associated with roasted or nutty notes [45]. The data support CSM and RSM as viable SBM alternatives without detriment to meat chemical composition, while indicating that CSM may enrich selected flavor-relevant AA pools under nutritionally balanced conditions [40].

### 4.3. Metabolites in Fresh LT Samples

In the current study, the untargeted metabolomics of fresh LT revealed broadly conserved muscle metabolic profiles across diets, as PCA and PERMANOVA did not detect global separation among SBM, CSM, and RSM treatments, meaning that most of metabolites in meat were unaffected. A small set of differential metabolites were identified by volcano plot filtering (FC > 2; FDR < 0.05), indicating diet-related, pathway-localized shifts rather than extensive metabolic remodeling. Specifically, galactose and 13-octadecenoic acid were lower in both CSM and RSM compared to SBM, while RSM had higher L-pyridosine and phenylalanyl-alanine than SBM; mannose-6-phosphate was higher in CSM than in RSM, which are relevant as they map to carbohydrate-derived, lipid-associated, and N-related substrate pools that are influenced by breed, diet composition, and postmortem handling [46], contributing to Maillard/Strecker chemistry and lipid oxidation during cooking [47,48].

The higher levels of mannose 6-phosphate in the CSM group may lead to a richer aroma of grilled meat after cooking. Meinert et al. (2009) found that mannose contributes to the formation of the "grilled meat aroma" in fried pork chops, and the contribution of glucose 6-phosphate is greater in enhancing meat flavor than glucose monosaccharides [48]. Galactose were markedly reduced in both RSM and CSM groups compared to SBM for reasons that replacing SBM with RSM can lower muscle carbohydrate intermediates in piglets [49] and reflect diet-driven differences in rumen fermentation and post-absorptive carbohydrate handling [32,50]. Water-soluble carbohydrates, including glucose, galactose, mannose, and their phosphorylated derivatives, are key substrates for Maillard reactions and Strecker degradation, serving as critical precursors for the formation of heterocyclic compounds, aldehydes, and ketones during thermal processing [47,48]. 

Increased phenylalanyl-alanine and L-pyridosine in the RSM group could contribute to taste attributes such as bitterness individually [51], bringing a bad taste to consumers, because phenylalanyl were bitter amino acids and L-pyridosine originated from bitter amino acids of lysine under thermal or chemical conditions. L-pyridosine is related to regulating amino acid transamination and nitrogen metabolism, which will potentially influence precursor availability for Strecker-derived aldehydes [47]. Increased phenylalanyl-alanine reflect subtle differences in peptide pools originating from postmortem proteolysis.

The contribution of monounsaturated fatty acids to meat flavor and taste is not significant. The lower 13-octadecenoic acid (a C18:1 feature) may represent a positional or geometric isomer within the broader rumen biohydrogenation-derived C18:1 pool in ruminants, where multiple C18:1 isomers arise from microbial biohydrogenation and respond to dietary lipid–protein contexts [52]. 

The metabolomic signature suggests that replacing SBM with CSM or RSM may modestly shift the balance between carbohydrate- and nitrogen-associated precursors while also altering a small subset of lipid-related features. However, the small effect size at the global level is consistent with the largely unchanged cooked-volatile outcomes, underscoring that precursor abundance is only one determinant of aroma generation, along with reaction kinetics, pH, water activity, and oxidative environment during heating.

### 4.4. Volatile Compounds of Cooked LT Samples

Results show that substituting SBM with CSM or RSM did not result in pronounced changes the overall volatile profile, suggesting that modest diet-related shifts in precursor metabolites in fresh muscle may be masked during cooking when process drivers (pH, water activity, heating rate, and oxygen exposure) dominate reaction pathways and volatile yields. Consistent with this chemistry, ANOVA detected no differences among diets in either major volatile classes or individual compounds, suggesting that protein-source replacement under comparable dietary energy and lipid supply have a limited influence on volatiles. Across treatments, hydrocarbons, alcohols, aldehydes, and ketones, which largely arise from lipid oxidation and Maillard–lipid interactions, were the major classes detected in cooked meat [53]. The stability of lipid oxidation-derived aldehydes (e.g., octanal, (E)-2-nonenal, and 2,4-nonadienal) was also consistent with the unchanged muscle FA profile, which aligns with evidence that lamb flavor is closely related to lipid composition and energy density, while protein source has smaller effects unless it alters lipid substrates [45].

In addition, 1-octen-3-ol and sulfur-containing compounds (notably dimethyl trisulfide) consistently that ranked among the odorants with higher ROAV values in a set of relatively important odor profile were predicted by ROAV analysis, as reported previously for Tan lamb [24,25,54], inferred that these two volatiles may be special to pooled LT of Tan lamb. 1-Octen-3-ol is typically linked to oxidative pathways and remained abundant across diets, indicating comparable conditions for lipid-derived odorant formation, can endow meat with mushroom, fermented flavor and earthy notes [55]. Sulfur volatiles, including dimethyl disulfide, dimethyl trisulfide, and 3-(methylthio)propanal, are derived from Maillard and Strecker chemistry involving reducing sugars and sulfur donors (e.g., Met), yet their prominence did not increase in parallel with the higher Met concentration in fresh muscle of CSM-fed lambs, indicating that sulfur odorant yield may be influenced by the broader reaction network, availability of reducing sugars and reactive carbonyls, redox environment, and thermal kinetics, as well as by precursor abundance alone [53]. The dominant dimethyl disulfide gives the meat a sulfur, onion, and cooked-meat aroma, which can increase consumer pleasure when consumed. High-ROAV lipid-oxidation products from Arachidonic acid, such as (E)-2-nonenal (fatty, paper) and (E,E)-2,4-decadienal (plastic, tailing odor), remained prominent across treatments, imparting grassy and fatty aroma to meat [21,53]. 

Overall, the odor-active volatile compounds (*E*)-2-nonenal, (*E*)-2-octenal, octanal, nonanal, (*E*,*E*)-2,4-decadienal, hexanal, heptanal, 1-octen-3-one, 1-octen-3-ol, 2-pentyfuran, methanethiol, dimethyl trisulfide, 2-acetyl-2-thiazoline, 2-pentylpyridine, and γ-nonalactone were the dominant contributors to the lamb meat odor profile. 

## 5. Conclusions

Replacing soybean meal (SBM) with cottonseed meal (CSM) and rapeseed meal (RSM) has no notable impact on lamb flavor. This is attributed to minor variations in reducing sugars, amino acids, fatty acids, and other substrates for Maillard reaction and lipid oxidation. The short experimental duration may also be a reason. The declined b* values of lamb in CSM and RSM groups may reduce shelf life, which deserves improvement in subsequent studies.

In addition, these results obtained by GC-MS allow us to infer that altering protein sources produces only limited changes in lamb muscle metabolites and flavor based on chemical data and the reported literature, but it remains unknown whether such changes objectively influence sensory evaluations of lamb—a major limitation of this study. Because analysis via GC-O-MS is commonly recognized as more accurate, future studies should consider integrating GC-O-MS technology for precise evaluation of OAV/ROAV.

## Figures and Tables

**Table 1:** Ingredients and nutritional level of experimental diets (DM basis).

Items	Groups		
	SBM	CSM	RSM
Ingredients, %			
Corn stalk	30	30	30
Corn	35	35	36
DDGS ^1^	10	10	10
Wheat bran	10	9	10
Soybean meal	5.5	-	-
Cottonseed meal	-	8	-
Rapeseed meal	-	-	5
Corn bran	5	4	4
Extruded urea	0.5	-	1
Premix ^2^	1	1	1
Limestone	1	1	1
CaHPO4	1	1	1
NaCl	1	1	1
Total	100	100	100
Nutritional level			
ME, MJ/kg ^3^	15.8	15.3	15.6
DM, %	91.1	90	91.3
CP, %	12.6	12.3	12.9
EE, %	1.8	1.8	1.8
NDF, %	43.4	42.1	47.9
ADF, %	23.5	24.4	25.5
Ca, %	0.82	0.85	0.97
P, %	0.2	0.22	0.22

ME = metabolizable energy; DM = dry matter; CP = crude protein; EE = ether extract; NDF = neutral detergent fiber; ADF = acid detergent fiber; ^1^ DDGS = distillers dried grains with solubles. ^2^ Premix provided the following per kg of diets: vitamin A (VA) 15,000 IU (International Units, 1 IU = 0.3 μg), vitamin D (VD) 2200 IU (1IU = 0.025 μg), vitamin E 50 IU (1 IU = 0.671 mg), Fe 55 mg, Cu 12.5 mg, Mn 47 mg, Zn 24 mg, Se 0.5 mg, and Co 0.1 mg. ^3^ ME (MJ/kg) was calculated as ME intake (MJ/d)/DMI (kg/d) during the digestibility trial.

**Table 2:** Growth performance of lambs fed diets with different protein sources.

Traits *	Groups ^1^			SEM	*p*-Value
	SBM	CSM	RSM		
IBW, kg	23.2	23.1	22.9	0.24	0.972
FBW, kg	41.8	40.9	41.8	0.24	0.136
DMI, kg/d	1.45	1.41	1.42	0.01	0.378
ADG, g/d	275.5	277.0	296.4	4.49	0.103
FCR	5.79	5.68	5.32	0.09	0.096

* Values are presented as means (n = 16 per group). IBW, initial body weight; FBW, final body weight; DMI, dry matter intake; ADG, average daily gain; FCR, feed conversion ratio; SEM, standard error of the mean. ^1^ Lambs received either TMR (Total Mixed Ration) composited with soybean meal (SBM), cottonseed meal (CSM), or rapeseed meal (RSM) as the protein source.

**Table 3:** Effects of dietary protein sources on the meat quality of LT in finishing sheep.

Traits	Groups ^1^			SEM	*p*-Value
	SBM	CSM	RSM		
Moisture, %	71.54	63.44	71.33	3.00	0.666
Ash, %	1.17	1.07	1.13	0.03	0.217
IMF ^2^, %	3.12	3.21	3.31	0.17	0.915
Protein, %	23.91	23.33	23.39	0.24	0.573
L* (45 min)	32.41	34.34	34.03	0.50	0.242
a* (45 min)	12.66	13.77	13.31	0.30	0.325
b* (45 min)	2.15 ^b^	3.12 ^a^	2.96 ^a^	0.17	0.034
Cooking loss, %	33.75	32.41	34.61	0.47	0.140
SF ^3^, N	43.1	44.87	43.12	1.82	0.903

^1^ Values are presented as means (n = 8 per group). ^2^ IMF = instrumental muscle fat; ^3^ SF = shear force; L* = lightness; a* = redness; b* = yellowness. Different superscript letters (a, b) within the same row indicate a significant difference (*p* < 0.05).

**Table 4:** Effects of dietary protein sources on fatty acid profiles of LT in finishing lambs.

FA ^1^ (mg/100 g)	Groups			SEM	*p*-Value
	SBM	CSM	RSM		
C10:0	0.69	0.44	0.64	0.07	0.297
C12:0	0.76	0.39	0.56	0.09	0.222
C14:0	13.81	8.40	10.82	1.46	0.332
C14:1	0.61	0.31	0.52	0.08	0.268
C15:0	2.12	1.42	2.50	0.31	0.371
C16:0	100.00	72.55	82.36	9.15	0.483
C16:1	10.21	6.89	8.85	0.94	0.367
C17:0	4.81	3.45	5.15	0.57	0.455
C18:0	51.87	43.18	42.24	4.71	0.674
C18:1n9t	21.10	18.53	15.99	2.85	0.780
C18:1n9c	149.60	114.29	126.81	12.47	0.524
C18:2n6c	27.03	25.53	25.03	1.87	0.910
C18:3n6	0.24	0.20	0.27	0.02	0.416
C20:1	0.27	0.18	0.29	0.04	0.591
C18:3n3	1.01	0.68	0.85	0.08	0.283
C20:2	0.13	0.13	0.13	0.03	0.991
C20:3n6	0.70	0.59	0.60	0.03	0.241
C20:4n6	8.59	7.68	7.59	0.30	0.343
C24:0	0.32	0.25	0.24	0.05	0.793
C22:6n3	0.19	0.24	0.26	0.03	0.699
SFA ^2^	174.39	130.08	144.50	15.98	0.534
MUFA ^3^	181.78	140.20	152.45	16.04	0.575
PUFA ^4^	37.89	35.05	34.74	2.18	0.822
n-3 PUFA ^5^	1.20	0.93	1.11	0.10	0.532
n-6 PUFA ^6^	36.57	33.99	33.49	2.08	0.824
n-6/n-3 PUFA	35.23	40.02	33.03	2.74	0.589

^1^ FA: fatty acid. ^2^ SFA: saturated fatty acids. ^3^ MUFA: monounsaturated fatty acids (sum of C14:1, C16:1, C18:1n9t, C18:1n9c, C20:1). ^4^ PUFA: polyunsaturated fatty acids (sum of C18:2n6c, C18:3n3, C18:3n6, C20:3n6, C20:4n6, and C22:6n3). ^5^ n-3 PUFA: sum of C18:3n3, and C22:6n3. ^6^ n-6 PUFA: sum of C18:2n6c, C18:3n6, C20:3n6, C20:4n6.

**Table 5:** Effects of dietary protein sources on amino acid profiles of LT in finishing Tan lambs.

AA ^1^ (mg/100 g)	Group			SEM	*p*-Value
	SBM	CSM	RSM		
Asp	1.90	2.05	2.02	0.03	0.099
Thr	0.96	0.99	0.98	0.02	0.813
Ser	0.71	0.72	0.71	0.01	0.849
Glu	2.89	3.05	2.93	0.06	0.554
Gly	0.95	0.96	0.97	0.01	0.769
Ala	1.12	1.21	1.16	0.02	0.074
Val	0.99	1.03	1.02	0.01	0.312
Met	0.51 ^b^	0.6 ^a^	0.55 ^ab^	0.01	0.044
Ile	1.04	1.10	1.08	0.01	0.139
Leu	1.61	1.70	1.67	0.02	0.177
Tyr	0.62	0.67	0.65	0.01	0.060
Phe	0.83	0.85	0.84	0.01	0.722
Lys	2.01	2.1	2.07	0.03	0.400
His	0.72	0.74	0.72	0.01	0.808
Arg	0.97	0.87	0.89	0.04	0.563
Pro	1.34	1.42	1.39	0.02	0.164
TAA ^2^	19.16	20.06	19.66	0.27	0.421
EAA ^3^	8.67	9.1	8.93	0.11	0.307
FAA ^4^	9.69	10.22	9.99	0.14	0.333
NEAA ^5^	10.49	10.95	10.72	0.16	0.525
EAA/TAA	0.45	0.45	0.45	0.00	0.775
EAA/NEAA	0.83	0.83	0.83	0.00	0.781

^1^ AA: amino acid. ^2^ TAA: total amino acids. ^3^ EAA: essential amino acids (sum of His, Ile, Leu, Lys, Met, Phe, Thr, and Val). ^4^ FAA: flavor amino acids (sum of Ala, Asp, Glu, Gly, Phe, and Lys). ^5^ NEAA: sum of Ala, Arg, Asp, Cys, Glu, Gln, Gly, Pro, Ser, and Tyr. Different lowercase letters indicate significant differences (*p* < 0.05); ^a^ represents the maximum value, ^b^ represents the middle value.

## Data Availability

The datasets used and analyzed in the current study are available from the corresponding author on reasonable request.

## Supplementary Materials

The following supporting information can be downloaded at: https://www.mdpi.com/article/10.3390/metabo16060387/s1, Table S1: Metabolites in fresh meat among three groups; Table S2: Contents of volatile classes and compounds among the three groups; Table S3: ANOVA test results of volatile compounds among three groups; Table S4: ROAV values of volatile compounds among three groups.

## Abbreviations

The following abbreviations are used in this manuscript:

VOC	Volatile compound
RSM	Rapeseed meal
SBM	Soybean meal
CSM	Cottonseed meal
DDGS	Distillers dried grains with solubles
DM	Dry matter
CP	Crude protein
EE	Ether extract
ME	Metabolizable energy
LT	Longissimus thoracic
TMR	Total mixed ration
ADF	Acid detergent fiber
NDF	Neutral detergent fiber
DMI	Dry matter intake
IBW	Initial body weight
FBW	Final body weight
FCR	Feed conversion ratio
ADG	Average daily gain
L*	Lightness
a*	Redness
b*	Yellowness
SFA	Saturated fatty acids
PUFA	Polyunsaturated fatty acids
MUFA	Monounsaturated fatty acids
n-3PUFA	Sum of C18:3n3 and C22:6n3
n-6PUFA	Sum of C18:2n6c C18:3n6, C20:3n6, and C20:4n6
TAA	Total amino acids
FAA	Flavor amino acids
EAA	Essential amino acids
E-nose	Electronic nose
FDR	False discovery rate
FC	Fold change
PCA	Principal component analysis
DFMs	Differential feature metabolites
ROAV	Relative odor activity value
